# Coping Behaviors and Psychological Disturbances in Youth Affected by the COVID-19 Health Crisis

**DOI:** 10.3389/fpsyg.2021.565657

**Published:** 2021-03-22

**Authors:** Mireia Orgilés, Alexandra Morales, Elisa Delvecchio, Rita Francisco, Claudia Mazzeschi, Marta Pedro, José Pedro Espada

**Affiliations:** ^1^Health Psychology Department, Universidad Miguel Hernández, Elche, Spain; ^2^Department of Philosophy, Social Sciences and Education, Università degli Studi di Perugia, Perugia, Italy; ^3^Católica Research Centre for Psychological - Family and Social Wellbeing, School of Human Sciences, Universidade Católica Portuguesa, Lisbon, Portugal

**Keywords:** quarantine, COVID-19, coping, stress, youth

## Abstract

The COVID-19 pandemic and the quarantine undergone by children in many countries is a stressful situation about which little is known to date. Children and adolescents' behaviors to cope with home confinement may be associated with their emotional welfare. The objectives of this study were: (1) to examine the coping strategies used out by children and adolescents during the COVID-19 health crisis, (2) to analyze the differences in these behaviors in three countries, and (3) to examine the relationship between different coping modalities and adaptation. Participants were 1,480 parents of children aged 3–18 years from three European countries (*n*_Spain_ = 431, *n*_Italy_ = 712, and *n*_Portugal_ = 355). The children's mean age was 9.15 years (*SD* = 4.27). Parents completed an online survey providing information on symptoms and coping behaviors observed in their children. The most frequent coping strategies were accepting what is happening (58.9%), collaborating with quarantine social activities (e.g., drawings on the windows, supportive applauses) (35.9%), acting as if nothing is happening (35.5%), highlighting the advantages of being at home (35.1%), and not appearing to be worried about what is happening (30.1%). Compared to Italian and Spanish children, Portuguese children used a sense of humor more frequently when their parents talked about the situation. Acting as if nothing was happening, collaborating with social activities, and seeking comfort from others were more likely in Spanish children than in children from the other countries. Compared to Portuguese and Spanish children, Italian children did not seem worried about what was happening. Overall, an emotional-oriented coping style was directly correlated with a greater presence of anxious symptoms, as well as to mood, sleep, behavioral, and cognitive alterations. Task-oriented and avoidance-oriented styles were related to better psychological adaptation (considered a low presence of psychological symptoms). Results also show that unaffected children or children with a lower level of impact were more likely to use strategies based on a positive focus on the situation. This study provides interesting data on the strategies to be promoted by parents to cope with the COVID-19 health crisis in children.

## Introduction

In the first quarter of 2020, a serious public health crisis emerged worldwide due to the spread of the severe acute respiratory syndrome coronavirus (SARS-CoV-2) or COVID-19 (World Health Organization (WHO), 2020). To control the spread of the coronavirus, measures to reduce social contact were implemented in many countries, varying from only social limitation to voluntary or mandatory confinement. Spain and Italy, with, respectively, more than 28,700 and 32,700 deaths as of May 25 (European Centre for Disease Prevention Control, 2020) were among the most affected countries worldwide. Other countries such as Portugal managed to reduce the impact in the number of infections and deaths, reaching a comparatively low figure of 1,316 deaths on the same date. It is paradoxical that, in Portugal, confinement was voluntary, whereas, in Italy and Spain, where infections and deaths were much higher, mandatory confinement was imposed.

Although there is a history of epidemics in the past, this is the first time in our recent history that confinement has been imposed to control a pandemic. However, few available studies confirm the psychological impact of confinement due to COVID-19 on children and adolescents. In a sample of 2,330 school-age children in Hubei province, China, 23% reported depressive symptoms and 19% reported anxiety symptoms after 34 days of confinement, a higher prevalence than that found in previous studies (Xie et al., 2020). Orgilés et al. (2020), in a sample of 1,143 parents of Italian and Spanish children aged 3–18 years, found that 86% of them observed changes in their children's emotional or behavioral state compared to before the quarantine. When comparing the impact of confinement on the young population of Italy, Spain, and Portugal in a study that involved parents of 1,480 children and adolescents aged 3–18 years, Francisco et al. (2020) concluded that children from Italy were less affected than those from Spain, but also surprisingly less affected than children from Portugal, the country where the rules for confinement had been less restrictive. The authors conclude that, although Portuguese children could go outdoors and enjoyed a more normalized life, observing different behavior patterns in each family could be confusing and worrisome for them. Italian children, with mandatory confinement but allowed to go outdoors (a short walk with one adult near their home), were better adapted than Spanish and Portuguese children.

Confinement has brought about a major change in the lives of children and adolescents. School closure has changed their academic routines, social distancing has limited their social relationships with their family members, and the closing of the public spaces has modified their leisure, restricting it to their home. During any confinement, there are numerous stressors. Brooks et al. (2020), through a review of 24 studies, highlight that the main stressors of confinement are long duration, fear of infection, frustration and boredom, and the lack of adequate information from health authorities. However, the effect of the stressors of a confinement situation on people's well-being could depend on how they cope with the situation.

Coping behaviors can be defined as intentional and conscious responses to the demands and emotions of stressful events (Lazarus, 1999; Compas et al., 2001). One of the most widely accepted classifications of coping behaviors is that of Lazarus and Folkman (1984), who distinguished between problem-focused and emotion-focused coping, as responses aimed at managing the problem that causes discomfort, and as regulating emotional responses to the problem, respectively. Subsequently, Parker and Endler (1992) observed that problem-focused coping strategies were associated with task orientation, whereas emotion-focused coping reflected an individual-focused orientation (Stanisławski, 2019). They included a third dimension called avoidance-oriented coping, which involved both task-focused and individual-focused strategies. The former is conceived as distraction (performing alternative tasks as a coping strategy), whereas the latter implies social amusement (instead of confronting the stressful situation). The ability to cope with stressful events and regulate emotions can play an important role in the explanation of why some children develop psychopathological symptoms while others not (Compas et al., 2017).

There is extensive literature on the behaviors that children and adolescents carry out to face stressful situations. Identifying coping behaviors in stressful situations can facilitate early effective interventions to reduce the risk of future psychological problems. Numerous studies have examined how certain coping behaviors can help to reduce stress whereas others have been associated with negative psychological symptoms. Specifically, coping behaviors of children and adolescents suffering from chronic diseases (e.g., Compas et al., 2012; Jaser et al., 2017), sexual abuse or mistreatment (e.g., Bal et al., 2009; Flett et al., 2012), war or post-war situations (e.g., Benson et al., 2011; Braun-Lewensohn et al., 2011), alcohol use (e.g., Catanzaro and Laurent, 2004), cancer (e.g., Compas et al., 2017), problems or conflicts between parents (e.g., Nicolotti et al., 2003; Shelton and Harold, 2008), terrorism (e.g., Rhoades et al., 2007), and natural disasters (e.g., Vigna et al., 2010; Zhang et al., 2010) among others, have been studied.

Psychological symptoms and behavioral changes in children and adolescents from European countries, including Italy, Spain, and Portugal, during the COVID-19 quarantine, have been described from the results of cross-sectional studies (Espada et al., 2020; Francisco et al., 2020; Orgilés et al., 2020; Xie et al., 2020). These studies conclude that a significant percentage of children have presented emotional and behavioral symptoms associated with the quarantine, and that a lower percentage of children have healthy habits (in terms of exercise, use of screens, and sleep patterns), compared to before the COVID-19 confinement. These findings are especially valuable to understand the impact of the early stages of the pandemic on children's well-being but the coping strategies they used to adapt to the pandemic have not yet been explored. Although much is known about children and adolescents' (coping strategies in numerous stressful situations, to date, little is known about how they are coping with the COVID-19 situation. As coping behaviors may significantly mediate children's mental health when facing the stress derived from the pandemic, knowing children and adolescents' specific coping strategies that are more closely related to psychological well-being would be very useful to prevent problems and intervene early in cases of risk. The objectives of this study were: (1) to examine the coping behaviors carried out by children and adolescents during the COVID-19 health crisis; (2) to analyze the differences in coping behaviors in children from Spain, Italy, and Portugal; and (3) to examine the relationship between different coping strategies and adaptation to home confinement.

## Method

### Participants

Participants were parents of 1,480 parents from three European countries (Italy *n* = 712, Spain *n* = 431, and Portugal *n* = 335). The average age of the participating parents was 42.26 years (*SD* = 5.92) and 87.8% were females. Sixty percent reported earning from 1,000 to 2,999 euros monthly, 7.4% earned 5,000 or more, and only 6.6% earned less than 1,000 euros. That is, the sample was predominantly middle class. Children were aged between 3 and 18 years (*M* = 9.15, *SD* = 4.27), and 47.2% were females. Participants were equivalent across countries in all sociodemographic variables, except for going outdoors, the number of people living at home, square meters per home, and children's age. Italian participants were more likely to have a garden than Spanish participants. The Spanish sample was more likely than the Portuguese sample to have a terrace. Participants from Italy and Portugal reported having a higher average number of people living at home during the confinement. Portuguese participants had larger homes than the Spaniards and Italians. Portuguese children were slightly older than the Spanish children. Table 1 describes the characteristics of the sample and differences across countries.

**Table 1:** Sample characteristics and equivalence by country.

	**Total (*n* = 1,480)**	**Italy (1) (*n* = 712)**	**Spain (2) (*n* = 431)**	**Portugal (3) (*n* = 335)**	**Test^**a**^**	**Effect size^**b**^**	***Post-Hoc* ^**c**^**
**Parents**							
Female, *N* (%)	1,299 (87.8)	627 (88.1)	379 (87.9)	293 (86.9)	0.28	-	-
Age, *M* (*SD*)	42.26 (5.92)	42.38 (6.64)	42.17 (5.32)	42.10 (4.96)	2.68	-	-
**Monthly family income (euros)**, ***N*** **(%)**							
Up to 999	87 (6.6)	33 (5.3)	31 (8.3)	23 (7.3)	14.82	-	-
Between 1,000 and 1,999	372 (28.2)	164 (26.2)	113 (30.1)	95 (30.1)			
Between 2,000 and 2,999	417 (31.8)	209 (33.4)	98 (26.1)	110 (34.8)			
Between 3,000 and 4,999	343 (26)	169 (27)	106 (28.3)	68 (21.5)			
5,000 or more	98 (7.4)	51 (8.1)	27 (7.2)	20 (6.3)			
**The house where you live has**, ***N*** **(%)**							
Only windows	158 (10.7)	25 (3.5)	77 (17.9)	56 (16.6)	221.39^***^	0.27	2 > 1 3 > 1
Garden	559 (37.8)	368 (51.7)	77 (17.9)	114 (33.8)			1 > 2
Terrace	303 (20.5)	151 (21.1)	121 (28.1)	31 (9.2)			2 > 3
Balcony	416 (28)	141 (19.9)	145 (33.5)	130 (38.6)			2 > 1 3 > 1
Another exit	44 (3)	27 (3.8)	11 (2.6)	6 (1.8)			
**People who live in my house during quarantine**, ***N*** **(%)**							
They do not leave the house unless they have to buy groceries or other allowed activities	936 (63.1)	463 (65)	254 (58.9)	217 (64.4)	4.59	-	-
One or both parents still work outside the home	546 (36.9)	249 (35)	177 (41.1)	120 (35.6)			
How many people live in at home during quarantine, *M* (*SD*)	3.94 (0.94)	3.99 (0.97)	3.84 (0.88)	3.98 (0.95)	9.73^**^	0.007	1 > 2 3 > 2
Square meters home, *M* (*SD*)	131.04 (67.70)	123.14 (62.29)	124.99 (62.86)	152 (78.89)	46.80^***^	0.03	3 > 1 3 > 2
**Children**							
Female, *N* (%)	699 (47.2)	351 (49.3)	192 (44.5)	156 (46.3)	2.58	-	-
Age, *M* (*SD*)	9.15 (4.27)	9.40 (4.46)	8.55 (3.73)	9.42 (4.45)	8.58^*^	0.006	1 > 2

*Note. M, Mean; SD, Standard Deviation;*

a *Cross-table (χ^2^) for categorical variables and Kruskal-Wallis (χ^2^) for continuous variables*.

b *Effect size = Cramer's V for multi-categorical variables and Epsilon-squared for continuous variables*.

c *Bonferroni correction applied to p values was used to reduce the risk of type I errors post hoc analysis of a chi-squared test*.

* *p < 0.05*,

** *p < 0.01*,

*** *p < 0.001*.

### Procedure

Participants were recruited in each country *via* social media (Facebook, Twitter, Instagram, and WhatsApp), as face-to-face contact was not allowed. An online survey was created *ad hoc* and distributed using a snowball sampling strategy. The data collection period was the same in the three participating countries (lasting 15 days, with the study starting 15 days after the lockdown). Parents from 94 Italian cities, 94 Portuguese cities, and 84 Spanish cities participated in the study. To create the survey, scientific literature related to the psychological impact of quarantines was reviewed, and questionnaires applied in previous studies with adult population were considered. The survey collected sociodemographic information of parents and children (see Table 1), parental perception of how the quarantine affected their children emotionally, and parental perception of the coping strategies that their children used during the quarantine. The children's psychological responses to the quarantine were assessed through 31 symptoms on a scale ranging from 1 (*much less compared to before quarantine*) to 5 *(much more compared to before quarantine*) and grouped into six categories: anxiety symptoms (e.g., he/she is worried or is anxious), mood symptoms (e.g., he/she is sad or cries easily), sleep problems (e.g., he/she is afraid of sleeping alone or has nightmares), behavioral alterations (e.g., he/she often argues with other members of the family or is uneasy), feeding problems (he/she eats more or has no appetite), and cognitive alterations (e.g., he/she is very indecisive or has difficulty concentrating). As part of the same study, Francisco et al. (2020) previously explored psychological symptoms and behavioral changes in Spanish children and adolescents during the early phase of the COVID-19 confinement. Coping behaviors were measured using a list of eleven items that included the three dimensions proposed by Parker and Endler (1992): task-oriented (e.g., “Highlights the pros of being at home”), emotion-oriented (e.g., “Talks often about how he/she feels”), and avoidance-oriented (e.g., “Changes the subject when you try to talk to him/her about the coronavirus or quarantine”). Instead of a known measure, a specific list of statements was chosen to assess coping so that the content was specific to the COVID-19 context. Before completing the survey, information about the objectives of the study was provided, and informed consent was requested. The approval of the Ethics Board of the authors' institution was obtained for the research.

### Data Analyses

Descriptive statistics were used to describe the characteristics of the sample. The normal distribution of the data was tested using the Kolmogorov–Smirnov test. Nonparametric procedures were used because of the absence of normality (*p* < 0.05). A total of 31 children's immediate psychological reactions were evaluated. Parents who informed that their children presented any of these reactions to a greater extent during home confinement (compared to before this period) were coded as 1 (“affected”), whereas the rest was coded as 0 (“unaffected”). A new variable (level of disturbance) was created by adding the 31 dichotomous variables of symptoms (score ranging from 0 to 31). The continuous variable (level of disturbance) was categorized into four groups: “unaffected” (The children had not worsened in any of the symptoms), “low impact” (had worsened in 1–3 symptoms), “medium impact” (had worsened in 4–9 symptoms), and “high impact” (had worsened in 10 or more symptoms).

Kruskal–Wallis tests were performed to analyze differences in coping styles (task-oriented, emotion-oriented, and avoidance-oriented) across the four groups of impact, and Chi-squared tests to compare proportions of children using each coping strategy (yes/no) across the levels of impact (four categories). A *p*-value under 0.05 was considered a statistically significant difference. To reduce the risk of Type I errors in multiple-comparisons across groups, Bonferroni corrections were applied to *p*-values (Beasley and Schumacker, 1995). For continuous variables, Epsilon-squared (ε2) was used as an effect size, where small effect sizes range from 0.01 to <0.08, medium effect sizes range from 0.08 to <0.26, and large effect sizes range from ≥ 0.26. For categorical variables, Chi-squared *post hoc* tests using adjusted residuals were performed. The percentage of children using each coping strategy was calculated to determine which ones were most frequently used by preschool children (3–5 years), school-age children (6–12 years), and adolescents (13–18 years). Cramer's *V* was calculated as a measure of association between multi-categorical variables, and interpreted as follows: > 0.25 very strong, > 0.15 strong, > 0.10 moderate, > 0.05 weak, and > 0 none or very weak (Akoglu, 2018). The relationship between coping styles and different areas of impact (anxiety, mood, sleep, feeding, behavioral and cognitive alterations) was explored using Spearman correlations. All analyses were performed using SPSS 26 for Mac.

## Results

### Coping Strategies

Table 2 shows the proportion of children using coping strategies during the home confinement due to COVID-19. The most frequently used coping strategy was acceptance, with more than half of the parents reporting that their children use it (58.9%). Other commonly used coping strategies (at least 30% of the children) were collaborating with social activities such as drawings on windows or collective applauses (35.9%), ignoring the problem and acting as if nothing was happening (35.5%), highlighting the advantages of being at home (35.1%), seeking comfort from others (31%), and not showing concern about what was happening (30.1%). According to age, the most used coping strategies (more than 30% of parents reported that their children used them) were similar among preschool children, school-age children, and adolescents, although their order could differ for each group. In preschool children (3–5 years), the most used coping strategies were: accepts what's going on (45.5%) (Task-oriented); acts as if nothing is happening (44.4%) (Avoidance-oriented); doesn't seem to care what is happening (40%) (Avoidance-oriented); and seeks affection from others (36.9%) (Emotional-oriented). In the school-age children (6–12 years), the most used coping strategies were: accepts what's going on (60.6%) (Task-oriented); highlights the advantages of being at home (41.3%) (Task-oriented); seeks affection from others (33.8%) (Emotion-oriented); and acts as if nothing is happening (32.3%) (Avoidance-oriented). In the adolescent group (13–18 years), the most used strategies were: accepts what's going on (69.9%) (Task-oriented); highlights the advantages of being at home (37.9%) (Task-oriented); and acts as if nothing is happening (32.2%) (Avoidance-oriented). When comparing the three countries, and after applying for Bonferroni correction, Spanish children were more likely to collaborate in social activities than children from the other countries. Compared to the Italian children, those from Portugal were also more likely to collaborate in social activities. Spanish children were more likely to seek affection in others, compared to the rest of children. Italian children were more likely to act as if they were not worried about what was happening, compared to the rest. Compared to the Portuguese children, those from Spain were also more likely to seem worried about what is happening.

**Table 2:** Coping strategies by country.

	**Total (*****n*** **=** **1,480)**		**Italy (1) (***n*** = 712)**		**Spain (2) (***n*** = 431)**		**Portugal (3) (***n*** = 335)**		**Test^a^**	**Effect size^b^**	***Post-hoc***
	***N***	**%**	***n***	**%**	***n***	**%**	***n***	**%**			
**Task-Oriented strategies**											
Asks very often about coronavirus or quarantine	355	24	166	23.3	91	21.1	98	29.1	6.92^*^	0.06	–
Highlights the pros of being at home	519	35.1	234	32.9	156	36.2	129	38.3	3.28	–	–
Uses humor when you talk about quarantine or coronavirus	226	15.3	99	13.9	60	13.9	67	19.9	7.17^*^	0.07	3 > 1 3 > 2
Collaborates with social activities	531	35.9	183	25.7	217	50.3	131	38.9	72.58^***^	0.22	2 > 1 2 > 3 3 > 1
Accepts what's going on	872	58.9	400	56.2	273	63.3	199	59.1	5.92	–	–
**Emotion-Oriented strategies**										–	–
Often talks about how he/she feels	201	13.6	103	14.5	46	10.7	52	15.4	4.56	–	–
Says he/she is very angry about what is happening	220	14.9	121	17	64	14.8	35	10.4	7.89^*^	0.01	1 > 3
Seeks affection in others	459	31	199	27.9	167	38.7	93	27.6	17.01^***^	0.10	2 > 1 2 > 3
**Avoidance-Oriented strategies**											
Changes conversations when you try to talk to him/her about the coronavirus or quarantine	122	8.2	52	7.3	41	9.5	29	8.6	1.80	–	–
Acts as if nothing is happening	525	35.5	242	34	183	42.5	100	29.7	14.82^**^	0.10	2 > 3 2 > 1
Doesn't seem worried about what is happening	445	30.1	252	35.4	130	30.2	63	18.7	30.33^***^	0.14	1 > 3 2 > 3 1 > 2

a *Cross-table (χ^2^) for categorical variables*.

b *Effect size = Cramer's V for multi-categorical variables*.

Bonferroni correction applied to p values was used to reduce the risk of type I errors post hoc analysis of a chi-squared test (resulting p-value = 0.0015). Only ^***^p < 0.0015 was considered statistically significant after applying for Bonferroni correction. However, differences that were significant at ^*^p < 0.05 and ^**^p < 0.01 were also indicated in the table..

### Relationship Between Coping Strategies and Children's Immediate Psychological Responses

The psychological impact of the quarantine on children was measured through 31 symptoms grouped into six categories: anxiety symptoms, mood symptoms, sleep problems, behavioral alterations, eating problems, and cognitive alterations. Coping strategies were also grouped depending on whether they were task-, emotion-, or avoidance-oriented (Table 3).

**Table 3:** Coping strategies and psychological responses.

	**Total (*****n*** **=** **1,480)**	
	***M***	***SD***
**Coping strategies**		
Task-oriented strategies (range 0–5)	1.69	1.13
Emotion-Oriented (range 0–3)	0.59	0.76
Avoidance-Oriented (range 0–3)	0.74	0.83
**Symptoms** (range 1–5)		
Anxiety symptoms	2.64	2.53
Mood symptoms	1.72	1.62
Sleep problems	0.70	1.21
Behavioral alterations	1.51	1.62
Feeding problems	0.33	0.54
Cognitive alterations	0.36	0.61
Symptoms total (range 0–31)	7.25	6.10

*M, Mean; SD, Standard Deviation*.

Table 4 presents the Spearman correlations between task-oriented, emotion-oriented, and avoidance-oriented coping styles and different areas of impact (anxiety, mood, sleep, eating, behavioral and cognitive alterations). The use of a task-oriented coping style was related to fewer symptoms in general, and fewer symptoms related to mood, sleep, behavioral, and cognitive alterations. The relationships observed across these variables were small (ρ from −0.08 to −0.17). The use of an emotion-oriented coping style was related to a higher number of symptoms, more anxiety, mood disturbances, sleep, behavioral, and cognitive alterations. The relationships observed across variables were small (ρ from 0.12 to 0.28). The use of an avoidance-oriented coping style was related to fewer symptoms in all areas, including eating. The relationships observed across variables were small (ρ from −0.05, −0.20).

**Table 4:** Correlations with confidence intervals for coping strategies and child's immediate psychological responses.

**Psychological responses**	**Task-oriented coping strategies**	**Emotion-oriented coping strategies**	**Avoidance-oriented coping strategies**
Anxiety symptoms	−0.03[−0.08, 0.02]	0.28^**^[0.23, 0.32]	−0.20^**^[−0.25, −0.15]
Mood symptoms	−0.17^**^[−0.22, −0.12]	0.22^**^[0.17, 0.27]	−0.08^**^[−0.13, −0.03]
Sleep problems	−0.12^**^[−0.17, −0.07]	0.15^**^[0.10, 0.20]	−0.05^*^[−0.10, −0.00]
Behavioral alterations	−0.15^**^[−0.20, −0.10]	0.20^**^[0.15, 0.25]	0.00[−0.05, 0.05]
Feeding problems	−0.03[−0.08, 0.02]	−0.03[−0.08, 0.02]	0.06^*^[0.01, 0.11]
Cognitive alterations	−0.08^**^[−0.13, −0.03]	0.12^**^[0.07, 0.17]	0.01[−0.04, 0.06]
Symptoms total	−0.13^**^[−0.18, −0.08]	0.27^**^[0.22, 0.31]	−0.11^**^[−0.16, −0.06]

*Note. Values in square brackets indicate the 95% confidence interval for each correlation. The confidence interval is a plausible range of population correlations that could have caused the sample correlation (Cumming, 2014).*

* *indicates p < 0.05*.

** *indicates p < 0.01*.

### Relationship Between Coping Strategies and Children's Level of Disturbance

Children were classified depending on their level of disturbance: “unaffected” (the children had not worsened in any of the symptoms), “low impact” (had worsened in 1–3 symptoms), “medium impact” (had worsened in 4–9 symptoms), and “high impact” (had worsened in 10 or more symptoms). Table 5 shows the coping strategies used by children with different levels of disturbance. Of the 11 coping strategies, 8 were related to the level of disturbance. Children more psychological affected by home confinement were more likely to use the following strategies: asking very often about coronavirus or quarantine, saying they were very angry about what was happening, seeking affection from others, and changing the subject when others tried to talk to them about the coronavirus or quarantine. Children with a lower level of disturbance due to home confinement were more likely to highlight the advantages of being at home, accept what was happening, act as if nothing was happening, and not seem worried about what was happening. The coping strategies “he/she uses humor when you talk about quarantine or coronavirus,” “collaborates in social activities” and “often talks about how they feel” were unrelated to the level of disturbance due to home confinement in children's psychological reactions.

**Table 5:** Coping strategies based on the level of disturbance.

**Coping strategies**	**(0) No affected *n* = 186**	**(1) LowAffected*n* = 311**	**(2) Middle affected *n* = 501**	**(3)HighAffected*n* = 482**	**Test^*a*^**	**Effect size^*b*^**	***Pairwise***
**Task-Oriented**, ***N*** **(%)**							
Asks very often about coronavirus or quarantine	36 (19.4)	48 (15.4)	107 (21.4)	164 (34)	43.20^***^	0.17	3 > 0 3 > 1
Highlights the pros of being at home	72 (38.7)	131 (42.1)	199 (39.7)	117 (24.3)	37.30^***^	0.15	0 > 3 1 > 3
Uses humor when you talk about quarantine or coronavirus	24 (12.9)	58 (18.6)	80 (16)	64 (13.3)	5.21	–	–
Collaborates with social activities (drawings on the windows, applauses)	71 (38.2)	118 (37.9)	168 (33.5)	174 (36.1)	2.20	–	–
Accepts what's going on	110 (59.1)	212 (68.2)	337 (67.3)	213 (44.2)	68.60^***^	0.21	2 > 3 1 > 3
**Emotion-Oriented**, ***N*** **(%)**							
Often talks about how he/she feels	21 (11.3)	37 (11.9)	71 (14.2)	72 (14.9)	2.48	–	–
Says he/she is very angry about what is happening	23 (12.4)	20 (6.4)	53 (10.6)	124 (25.7)	70.60^***^	0.21	3 > 2 3 > 1
Seeks affection in others	40 (21.5)	58 (18.6)	161 (32.1)	200 (41.5)	55.12^***^	0.19	3 > 0 3 > 1
**Avoidance-Oriented**, ***N*** **(%)**							
Changes conversations when you try to talk to him/her about the coronavirus or quarantine	7 (3.8)	9 (2.9)	33 (2.2)	73 (15.1)	48.87^***^	0.18	3 > 0 3 > 1 3 > 2
Acts as if nothing is happening	81 (43.5)	129 (41.5)	173 (34.5)	142 (29.5)	18^***^	0.11	0 > 3 1 > 3
Doesn't seem worried about what is happening	74 (39.4)	119 (38.3)	148 (29.5)	104 (21.6)	34.88^***^	0.15	0 > 3 1 > 3

*Note.*

a *Cross-table (χ^2^) for categorical variables*.

b *Effect size = Cramer's V for multi-categorical variables*.

Bonferroni correction applied to p values was used to reduce the risk of type I errors post hoc analysis of a chi-squared test (resulting p-value = 0.0011). Only ^***^p < 0.0011 was considered statistically significant after applying for Bonferroni correction.

## Discussion

The objective of the present study was to examine for the first time the strategies used by children and adolescents to cope with the quarantine imposed by governments to control the COVID-19. Another objective was to study the relationship between children's coping strategies and their emotional and behavioral responses to the home confinement to determine which strategy is more useful to cope with the situation. Different rules of confinement were also analyzed, as three European countries participated in the study.

Results show that the most frequently used coping strategy was task-oriented (accepting what was happening), with 59% of parents reporting its use by their children. Also, at least 30% of the children collaborated in social activities, acted as if nothing was happening, highlighted the advantages of being at home, sought comfort from others, or did not seem worried about what was happening. Differences by countries show interesting results. Collaborating in social activities and seeking comfort from others were more likely in Spanish children than in children from the other countries. Compared to Portuguese and Spanish children, Italian children did not seem worried about what was happening. Although it is unclear, the different rules of confinement imposed by each country could explain these differences. Portugal followed voluntary confinement, so maybe children's routines did not change as much as in the other countries; the few cases of infections and deaths compared to Spain and Italy might have contributed to their not perceiving the situation as dangerous. Children from Spain used adaptive strategies to cope with the situation, such as collaborating in social activities, but they were also more likely to seek comfort from their parents. Spain had the most restrictive confinement rules, not allowing children to go outside until April 26th. Although more data are necessary to explain this finding, the interruption of all social contact and staying at home with the parents for such a long time could have encouraged Spanish children to seek more comfort than Portuguese and Italian children, who followed a less restrictive confinement. Also, Spanish children collaborated more in social activities, such as collective applauses from the balconies or windows, probably showing their need for social contact with others, which was limited indoors. Finally, Italian children seem less concerned about the situation than children from the other countries. Unlike Italy, Portugal used voluntary confinement, with habits and routines depending on each family's decision, so the children may have perceived inconsistent situations outdoors that might have worried them. Italian children were allowed to go outside before Spanish children, so Spanish children may have been more worried than Italian children because they had to follow the prohibition of going outside. Although further research is needed, allowing Italian children to go outside while maintaining consistent rules for all the children (a walk with one adult near their house) may have reduced their concerns.

A main objective of this study was to analyze the relationship between coping strategies and children's behavioral and emotional symptoms reported by parents. As there is a lack of studies examining how children cope with home confinement, we tried to explore which strategies were more related to children's well-being, and thus more useful for them to cope with the situation. Results show that children who use an emotion-oriented coping style have more behavioral and emotional symptoms (more anxiety, mood disturbances, sleep, behavioral and cognitive alterations). Contrarily, those who use a task-oriented or an avoidance-oriented coping strategy have fewer emotional and behavioral symptoms, specifically, fewer symptoms related to mood, sleep, behavioral and cognitive alterations. These results are in line with previous studies finding that emotion-focused coping, in which attention is not directed to solving the problem but to one's emotional experience (Sears et al., 2000), is usually related to internalizing symptoms, such as anxiety or mood, and externalizing symptoms, such as behavioral alterations (Carlo et al., 2012).

To define which strategy could be more useful to cope with the quarantine, children were classified as unaffected, low impact, medium impact, and high impact, as a function of the number of symptoms reported. Results show that unaffected children or children with a lower level of impact are more likely to highlight the advantages of being at home, accept what is happening, act as if nothing was happening, and not seem worried about what was happening. Although a unique type of coping strategy related to psychological symptoms has not been found in this study, these four strategies have in common a positive focus on the situation. Contrariwise, children considered more psychologically affected used one of these four specific strategies to cope with home confinement: asking very often about coronavirus or quarantine, feeling angry about what was happening, seeking comfort from other members of the family, and changing the subject when the parents tried to talk to them about the situation. These coping strategies have been related to psychopathological symptoms in previous studies. Avoidance and rumination have been frequently associated with anxiety and depression, whereas acceptance has shown a negative relationship with those symptoms (Schäfer et al., 2017). Asking frequently about the stressful situation may be considered as a strategy of rumination, which showed a high positive association with anxiety and depression symptoms, among other problems, in a meta-analysis that included studies carried out with children (Aldao et al., 2010). Avoidance had a small-to-medium positive association with anxiety and depression in that same meta-analytic review, supporting the finding of our study that indicate that changing the subject away from confinement is a typical coping strategy of psychologically affected children. Feeling angry or seeking comfort from others are also usual coping strategies shown by children for coping with distress (e.g., Miers et al., 2007; Zimmer-Gembeck and Skinner, 2011).

This study has some limitations and some strengths. The main limitation of this study is that the information was collected online from parents, as contact with the children was not possible due to the confinement situation. Despite the importance of applying self-reports, some previous studies have examined coping using measures completed by parents, as in the present study (e.g., Connor-Smith et al., 2000). In this study, children's coping strategies were evaluated, and these can be easily identified by people who live with the child (e.g., asks very often about coronavirus or quarantine or seeks affection from others). Therefore, parents were considered better informants than the children. Also, data collection *via* the parents allowed us to obtain information about younger children. Although it would have been desirable to use a multi-informant method, it was not possible due to the limited access to children during home confinement. Although the sample is not representative, it includes a large number of cities in three European countries (94 cities in Portugal, 94 in Italy, and 84 in Spain) and can be illustrative of the behavior of children and adolescents in the first weeks of the COVID-19 crisis. More studies are required to examine how coping behaviors developed during the health crisis are related to the onset of future psychopathology, especially through a longitudinal approach.

To our knowledge, this is the first study examining the coping strategies used by children during the quarantine for COVID-19. The following conclusions of the study may be highlighted. First, a task-oriented strategy was the most common in the sample, although some differences between countries were found in the strategies used by children to cope with the situation. Second, children who used a task-oriented or an avoidance-oriented coping strategy showed fewer emotional and behavioral symptoms. Third, children who coped with the situation using positive strategies, such as highlighting the advantages of being at home, were less emotionally and behaviorally affected. The present study has provided information about the specific coping behaviors in the COVID-19 confinement that can be protective factors against psychopathological symptoms. Therefore, coping behaviors related to less distubance should be promoted by educators and professionals in early interventions.

## Data Availability Statement

The raw data supporting the conclusions of this article will be made available by the authors, without undue reservation.

## Ethics Statement

The studies involving human participants were reviewed and approved by Órgano Evaluador de Proyectos de la Universidad Miguel Hernández de Elche. DPS.MO.01S.17.

## Author Contributions

MO designed the study and the survey. JE designed this study and wrote the draft of this article. AM managed and analyzed data. ED designed the Italian survey and collected data. CM collected data. RF designed the Portuguese survey and collected data. MP collected data. All the authors reviewed the draft and contributed to the final version of the manuscript.

## Conflict of Interest

The authors declare that the research was conducted in the absence of any commercial or financial relationships that could be construed as a potential conflict of interest.

## References

[B1] Akoglu H. (2018). User's guide to correlation coefficients. Turkish J. Emerg. Med. 18, 91–93. 10.1016/j.tjem.2018.08.001 PMC6107969 30191186

[B2] Aldao A. Nolen-Hoeksema S. Schweizer S. (2010). Emotion-regulation strategies across psychopathology: A meta-analytic review. Clin. Psychol. Rev. 30, 217–237. 10.1016/j.cpr.2009.11.004 20015584

[B3] Bal S. Crombez G. De Bourdeaudhuij I. Van Oost P. (2009). Symptomatology in adolescents following initial disclosure of sexual abuse: The roles of crisis support, appraisals and coping. Child Abuse Negl. 33, 717–727. 10.1016/j.chiabu.2008.11.006 19815274

[B4] Beasley T. M. Schumacker R. E. (1995). Multiple regression approach to analyzing contingency tables: *Post hoc* and planned comparison procedures. J. Exp. Educ. 64, 79–93. 10.1080/00220973.1995.9943797

[B5] Benson M. A. Compas B. E. Layne C. M. Vandergrift N. Pašalic H. Katalinksi R.. (2011). Measurement of post-war coping and stress responses: a study of Bosnian adolescents. J. Appl. Dev. Psychol. 32, 323–335. 10.1016/j.appdev.2011.07.001

[B6] Braun-Lewensohn O. Sagy S. Roth G. (2011). Coping strategies as mediators of the relationship between sense of coherence and stress reactions: Israeli adolescents under missile attacks. Anxiety Stress Coping Int. J. 24, 327–341. 10.1080/10615806.2010.494329 20582754

[B7] Brooks S. K. Webster R. K. Smith L. E. Woodland L. Wessely S. Greenberg N.. (2020). The psychological impact of quarantine and how to reduce it: rapid review of the evidence. Lancet 395, 912–920. 10.1016/S0140-6736(20)30460-8 32112714 PMC7158942

[B8] Carlo G. Mestre M. V. McGinley M. M. Samper P. Tur A. Sandman D. (2012). The interplay of emotional instability, empathy, and coping on prosocial and aggressive behaviors. Personal. Individ. Diff. 53, 675–680. 10.1016/j.paid.2012.05.022

[B9] Catanzaro S. J. Laurent J. (2004). Perceived family support, negative mood regulation expectancies, coping, and adolescent alcohol use: evidence of mediation and moderation effects. Addict. Behav., 29, 1779–1797. 10.1016/j.addbeh.2004.04.001 15530721

[B10] Compas B. E. Connor-Smith J. K. Saltzman H. Thomsen A. H. Wadsworth M. E. (2001). Coping with stress during childhood and adolescence: problems, progress, and potential in theory and research. Psychol. Bull. 127, 87–127. 10.1037/0033-2909.127.1.87 11271757

[B11] Compas B. E. Jaser S. S. Bettis A. H. Watson K. H. Gruhn M. A. Dunbar J. P.. (2017). Coping, emotion regulation, and psychopathology in childhood and adolescence: a meta-analysis and narrative review. Psychol. Bull. 143, 939–991. 10.1037/bul0000110 28616996 PMC7310319

[B12] Compas B. E. Jaser S. S. Dunn M. J. Rodriguez E. M. (2012). Coping with chronic illness in childhood and adolescence. Annu. Rev. Clin. Psychol. 8, 455–480. 10.1146/annurev-clinpsy-032511-143108 22224836 PMC3319320

[B13] Connor-Smith J. K. Compas B. E. Wadsworth M. E. Thomsen A. H. Saltzman H. (2000). Responses to stress in adolescence: measurement of coping and involuntary responses to stress. J. Consult. Clin. Psychol. 68, 976–992. 10.1037/0022-006X.68.6.976 11142550

[B14] Cumming G. (2014). The new statistics why and how. Psychol. Sci. 25, 7–29. 10.1177/0956797613504966 24220629

[B15] Espada J. P. Orgilés M. Piqueras J. A. Morales A. (2020). Buenas prácticas en la atención psicológica infanto-juvenil ante el COVID-19. Clín. Salud. 31:2020a14. 10.5093/clysa2020a14

[B16] European Centre for Disease Prevention Control (2020). Covid-19. Retrieved from https://qap.ecdc.europa.eu/public/extensions/COVID-19/COVID-19.html

[B17] Flett G. L. Druckman T. Hewitt P. L. Wekerle C. (2012). Perfectionism, coping, social support, and depression in maltreated adolescents. J. Ration. Emot. Cogn. Behav. Ther. 30, 118–131. 10.1007/s10942-011-0132-6

[B18] Francisco R. Pedro M. Delvecchio E. Espada J. P. Morales A. Mazzeschi C.. (2020). Psychological symptoms and behavioral changes in children and adolescents during the early phase of COVID-19 quarantine in three European countries. Front. Psychiatr. 11:570164. 10.3389/fpsyt.2020.570164 33343415 PMC7744455

[B19] Jaser S. S. Patel N. Xu M. Tamborlane W. V. Grey M. (2017). Stress and coping predicts adjustment and glycemic control in adolescents with Type 1 Diabetes. Ann. Behav. Med. 51, 30–38. 10.1007/s12160-016-9825-5 27496164 PMC5253083

[B20] Lazarus R. S. (1999). Hope: an emotion and a vital coping resource against despair. Soc. Res. 66, 653–678.

[B21] Lazarus R. S. Folkman S. (1984). Stress, Appraisal, and Coping. New York, NY: Springer.

[B22] Miers A. C. Rieffe C. Terwogt M. M. Cowan R. Linden W. (2007). The relation between anger coping strategies, anger mood and somatic complaints in children and adolescents. J. Abnorm. Child Psychol. 35, 653–664. 10.1007/s10802-007-9120-9 17554615

[B23] Nicolotti L. El-Sheikh M. Whitson S. M. (2003). Children's coping with marital conflict and their adjustment and physical health: vulnerability and protective functions. J. Fam. Psychol. 17, 315–326. 10.1037/0893-3200.17.3.315 14562456

[B24] Orgilés M. Morales A. Delvecchio E. Mazzeschi C. Espada J. P. (2020). Immediate psychological effects of the COVID-19 quarantine in youth from Italy and Spain. Front. Psychol. 11:579038. 10.31234/osf.io/qaz9w 33240167 PMC7677301

[B25] Parker J. D. A. Endler N. S. (1992). Coping with coping assessment: a critical review. Eur. J. Personal. 6, 321–344. 10.1002/per.2410060502

[B26] Rhoades G. K. McIntosh D. N. Wadsworth M. E. Ahlkvist J. A. Burwell R. A. Gudmundsen G. R.. (2007). Forgiving the September 11th terrorists: associations with coping, psychological distress, and religiosity. Anxiety Stress Coping 20, 109–128. 10.1080/10615800701195405 17999219

[B27] Schäfer J. O. Naumann E. Holmes E. A. Tuschen-Caffier B. Samson A. C. (2017). Emotion regulation strategies in depressive and anxiety symptoms in youth: a meta-analytic review. J. Youth Adolesc. 46, 261–276. 10.1007/s10964-016-0585-0 27734198

[B28] Sears S. F. Jr. Urizar G. G. Jr. Evans G. D. (2000). Examining a stress-coping model of burnout and depression in extension agents. J. Occup. Health Psychol. 5, 56–62. 10.1037/1076-8998.5.1.56 10658885

[B29] Shelton K. H. Harold G. T. (2008). Pathways between interparental conflict and adolescent psychological adjustment: bridging links through children's cognitive appraisals and coping strategies. J. Early Adolesc. 28, 555–582. 10.1177/0272431608317610

[B30] Stanisławski K. (2019). The coping circumplex model: an integrative model of the structure of coping with stress. Front. Psychol. 10:694. 10.3389/fpsyg.2019.00694 31040802 PMC6476932

[B31] Vigna J. F. Hernandez B. C. Kelley M. L. Gresham F. M. (2010). Coping behavior in hurricane-affected African American youth: psychometric properties of the Kidcope. J. Black Psychol. 36, 98–121. 10.1177/0095798408329948

[B32] World Health Organization (WHO). (2020). WHO director-general's opening remarks at the media briefing on COVID-19-11 March 2020. Retrieved from https://www.who.int/dg/speeches/detail/who-director-general-s-opening-remarks-at-the-media-briefing-on-covid-19-−11-march-2020.

[B33] Xie B. A. Qi Xue M. P. H. Yu Zhou B. A. Kaiheng Zhu B. A. Qi Liu M. S. Jiajia Zhang M. S.. (2020). Mental health status among children in home confinement during the coronavirus disease 2019 otubreak in Hubei Province, China. JAMA Pediatr. 174, 898–900. 10.1001/jamapediatrics.2020.1619 32329784 PMC7182958

[B34] Zhang Y. Kong F. Wang L. Chen H. Gao X. Tan X.. (2010). Mental health and coping styles of children and adolescents survivors one year after the 2008 Chinese earthquake. Child. Youth Ser. Rev. 32, 1403–1409. 10.1016/j.childyouth.2010.06.009

[B35] Zimmer-Gembeck M. J. Skinner E. A. (2011). The development of coping across childhood and adolescence: an integrative review and critique of research. Int. J. Behav. Dev. 35, 1–17. 10.1177/016502541038492

