# Corrigendum: Coping Behaviors and Psychological Disturbances in Youth Affected by the COVID-19 Health Crisis

**DOI:** 10.3389/fpsyg.2021.692133

**Published:** 2021-09-29

**Authors:** Mireia Orgilés, Alexandra Morales, Elisa Delvecchio, Rita Francisco, Claudia Mazzeschi, Marta Pedro, José Pedro Espada

**Affiliations:** ^1^Health Psychology Department, Universidad Miguel Hernández, Elche, Spain; ^2^Department of Philosophy, Social Sciences and Education, Università degli Studi di Perugia, Perugia, Italy; ^3^Católica Research Centre for Psychological - Family and Social Wellbeing, School of Human Sciences, Universidade Católica Portuguesa, Lisbon, Portugal

**Keywords:** quarantine, COVID-19, coping, stress, youth

In the original article, there was an error. All results that were statistically significant were informed, rather than only those that were found to be significant after applying the Bonferroni adjustment.

A correction has been made to *Results, Coping Strategies, Paragraph 1*. The corrected paragraph is shown below.

Table 2 shows the proportion of children using coping strategies during the home confinement due to COVID-19. The most frequently used coping strategy was acceptance, with more than half of the parents reporting that their children use it (58.9%). Other commonly used coping strategies (at least 30% of the children) were collaborating with social activities such as drawings on windows or collective applauses (35.9%), ignoring the problem and acting as if nothing was happening (35.5%), highlighting the advantages of being at home (35.1%), seeking comfort from others (31%), and not showing concern about what was happening (30.1%). According to age, the most used coping strategies (more than 30% of parents reported that their children used them) were similar among preschool children, school-age children, and adolescents, although their order could differ for each group. In preschool children (3–5 years), the most used coping strategies were: accepts what's going on (45.5%) (Task-oriented); acts as if nothing is happening (44.4%) (Avoidance-oriented); doesn't seem to care what is happening (40%) (Avoidance-oriented); and seeks affection from others (36.9%) (Emotional-oriented). In the school-age children (6–12 years), the most used coping strategies were: accepts what's going on (60.6%) (Task-oriented); highlights the advantages of being at home (41.3%) (Task-oriented); seeks affection from others (33.8%) (Emotion-oriented); and acts as if nothing is happening (32.3%) (Avoidance-oriented). In the adolescent group (13–18 years), the most used strategies were: accepts what's going on (69.9%) (Task-oriented); highlights the advantages of being at home (37.9%) (Task-oriented); and acts as if nothing is happening (32.2%) (Avoidance-oriented). When comparing the three countries, and after applying for Bonferroni correction, Spanish children were more likely to collaborate in social activities than children from the other countries. Compared to the Italian children, those from Portugal were also more likely to collaborate in social activities. Spanish children were more likely to seek affection in others, compared to the rest of children. Italian children were more likely to act as if they were not worried about what was happening, compared to the rest. Compared to the Portuguese children, those from Spain were also more likely to seem worried about what is happening.

Additionally, a correction has been made to *Discussion, Paragraph 2*.

Results show that the most frequently used coping strategy was task-oriented (accepting what was happening), with 59% of parents reporting its use by their children. Also, at least 30% of the children collaborated in social activities, acted as if nothing was happening, highlighted the advantages of being at home, sought comfort from others, or did not seem worried about what was happening. Differences by countries show interesting results. Collaborating in social activities and seeking comfort from others were more likely in Spanish children than in children from the other countries. Compared to Portuguese and Spanish children, Italian children did not seem worried about what was happening. Although it is unclear, the different rules of confinement imposed by each country could explain these differences. Portugal followed voluntary confinement, so maybe children's routines did not change as much as in the other countries; the few cases of infections and deaths compared to Spain and Italy might have contributed to their not perceiving the situation as dangerous. Children from Spain used adaptive strategies to cope with the situation, such as collaborating in social activities, but they were also more likely to seek comfort from their parents. Spain had the most restrictive confinement rules, not allowing children to go outside until April 26th. Although more data are necessary to explain this finding, the interruption of all social contact and staying at home with the parents for such a long time could have encouraged Spanish children to seek more comfort than Portuguese and Italian children, who followed a less restrictive confinement. Also, Spanish children collaborated more in social activities, such as collective applauses from the balconies or windows, probably showing their need for social contact with others, which was limited indoors. Finally, Italian children seem less concerned about the situation than children from the other countries. Unlike Italy, Portugal used voluntary confinement, with habits and routines depending on each family's decision, so the children may have perceived inconsistent situations outdoors that might have worried them. Italian children were allowed to go outside before Spanish children, so Spanish children may have been more worried than Italian children because they had to follow the prohibition of going outside. Although further research is needed, allowing Italian children to go outside while maintaining consistent rules for all the children (a walk with one adult near their house) may have reduced their concerns.

There were also errors in Tables 2 and 5 as published. The corrected Tables 2 and 5 are shown below.

**Table 2:** Coping strategies by country.

	**Total (*****n*** **=** **1,480)**		**Italy (1) (***n*** = 712)**		**Spain (2) (***n*** = 431)**		**Portugal (3) (***n*** = 335)**		**Test^a^**	**Effect size^b^**	***Post-hoc***
	***N***	**%**	***n***	**%**	***n***	**%**	***n***	**%**			
**Task-Oriented strategies**											
Asks very often about coronavirus or quarantine	355	24	166	23.3	91	21.1	98	29.1	6.92^*^	0.06	–
Highlights the pros of being at home	519	35.1	234	32.9	156	36.2	129	38.3	3.28	–	–
Uses humor when you talk about quarantine or coronavirus	226	15.3	99	13.9	60	13.9	67	19.9	7.17^*^	0.07	3 > 1 3 > 2
Collaborates with social activities	531	35.9	183	25.7	217	50.3	131	38.9	72.58^***^	0.22	2 > 1 2 > 3 3 > 1
Accepts what's going on	872	58.9	400	56.2	273	63.3	199	59.1	5.92	–	–
**Emotion-Oriented strategies**										–	–
Often talks about how he/she feels	201	13.6	103	14.5	46	10.7	52	15.4	4.56	–	–
Says he/she is very angry about what is happening	220	14.9	121	17	64	14.8	35	10.4	7.89^*^	0.01	1 > 3
Seeks affection in others	459	31	199	27.9	167	38.7	93	27.6	17.01^***^	0.10	2 > 1 2 > 3
**Avoidance-Oriented strategies**											
Changes conversations when you try to talk to him/her about the coronavirus or quarantine	122	8.2	52	7.3	41	9.5	29	8.6	1.80	–	–
Acts as if nothing is happening	525	35.5	242	34	183	42.5	100	29.7	14.82^**^	0.10	2 > 3 2 > 1
Doesn't seem worried about what is happening	445	30.1	252	35.4	130	30.2	63	18.7	30.33^***^	0.14	1 > 3 2 > 3 1 > 2

a *Cross-table (χ^2^) for categorical variables*.

b *Effect size = Cramer's V for multi-categorical variables*.

Bonferroni correction applied to p values was used to reduce the risk of type I errors post hoc analysis of a chi-squared test (resulting p-value = 0.0015). Only ^***^p < 0.0015 was considered statistically significant after applying for Bonferroni correction. However, differences that were significant at ^*^p < 0.05 and ^**^p < 0.01 were also indicated in the table..

**Table 5:** Coping strategies based on the level of disturbance.

**Coping strategies**	**(0) No affected *n* = 186**	**(1) LowAffected*n* = 311**	**(2) Middle affected *n* = 501**	**(3)HighAffected*n* = 482**	**Test^*a*^**	**Effect size^*b*^**	***Pairwise***
**Task-Oriented**, ***N*** **(%)**							
Asks very often about coronavirus or quarantine	36 (19.4)	48 (15.4)	107 (21.4)	164 (34)	43.20^***^	0.17	3 > 0 3 > 1
Highlights the pros of being at home	72 (38.7)	131 (42.1)	199 (39.7)	117 (24.3)	37.30^***^	0.15	0 > 3 1 > 3
Uses humor when you talk about quarantine or coronavirus	24 (12.9)	58 (18.6)	80 (16)	64 (13.3)	5.21	–	–
Collaborates with social activities (drawings on the windows, applauses)	71 (38.2)	118 (37.9)	168 (33.5)	174 (36.1)	2.20	–	–
Accepts what's going on	110 (59.1)	212 (68.2)	337 (67.3)	213 (44.2)	68.60^***^	0.21	2 > 3 1 > 3
**Emotion-Oriented**, ***N*** **(%)**							
Often talks about how he/she feels	21 (11.3)	37 (11.9)	71 (14.2)	72 (14.9)	2.48	–	–
Says he/she is very angry about what is happening	23 (12.4)	20 (6.4)	53 (10.6)	124 (25.7)	70.60^***^	0.21	3 > 2 3 > 1
Seeks affection in others	40 (21.5)	58 (18.6)	161 (32.1)	200 (41.5)	55.12^***^	0.19	3 > 0 3 > 1
**Avoidance-Oriented**, ***N*** **(%)**							
Changes conversations when you try to talk to him/her about the coronavirus or quarantine	7 (3.8)	9 (2.9)	33 (2.2)	73 (15.1)	48.87^***^	0.18	3 > 0 3 > 1 3 > 2
Acts as if nothing is happening	81 (43.5)	129 (41.5)	173 (34.5)	142 (29.5)	18^***^	0.11	0 > 3 1 > 3
Doesn't seem worried about what is happening	74 (39.4)	119 (38.3)	148 (29.5)	104 (21.6)	34.88^***^	0.15	0 > 3 1 > 3

*Note.*

a *Cross-table (χ^2^) for categorical variables*.

b *Effect size = Cramer's V for multi-categorical variables*.

Bonferroni correction applied to p values was used to reduce the risk of type I errors post hoc analysis of a chi-squared test (resulting p-value = 0.0011). Only ^***^p < 0.0011 was considered statistically significant after applying for Bonferroni correction.

The authors apologize for these errors and state that they do not change the scientific conclusions of the article in any way. The original article has been updated.

## Publisher's Note

All claims expressed in this article are solely those of the authors and do not necessarily represent those of their affiliated organizations, or those of the publisher, the editors and the reviewers. Any product that may be evaluated in this article, or claim that may be made by its manufacturer, is not guaranteed or endorsed by the publisher.

